# Evaluation of stress and coping skills of nursing students during a COVID-19 pandemic

**DOI:** 10.1192/j.eurpsy.2022.1248

**Published:** 2022-09-01

**Authors:** H. El Kefi, W. Kabtni, W. Krir, A. Baatout, I. Bouzouita, O. Torkhani, I. Gafsi, C. Bencheikh, A. Oumaya

**Affiliations:** Hmpit, Psychiatry, Tunis, Tunisia

**Keywords:** nurse student, stressors, Coronavirus, coping

## Abstract

**Introduction:**

The coronavirus epidemic started in Tunisia in March 2020. Health personnel have been in the front line in the fight against this virus since that date. The COVID units of the hospitals and the different hospital services have been reinforced by student nurses (trainees, volunteers…).

**Objectives:**

To evaluate the degree of stress perceived during the COVID-19 pandemic by student nurses. To identify coping skills during a COVID-19 pandemic.

**Methods:**

Descriptive, retrospective study conducted in March 2021 on the 60 senior nursing students enrolled in the military health school. We used the Cungi (1997) stress scale and developed a self-questionnaire on coping skills used by the students.

**Results:**

Our population was 54.3% male and 45.7% female. The average age was 22.6 years. The majority of the senior students (54.3%) worked in units dedicated to the care of patients with COVID-19. On the Cungi Stress Rating Scale, students had very low (13%), low (27%), and high (60%) stress levels. The main coping methods used were watching movies and listening to music (81%), playing sports (80%), praying (75%), rigorously applying social distancing measures and wearing protective gear (73%), talking with friends or psychologists (62%), doing yoga or meditation (34%), drinking herbal tea, alcohol, or taking psychotropic drugs (23%).

**Conclusions:**

The COVID-19 pandemic is a time of major stress for nursing students. The coping methods used seem insufficient. Psychological support should be provided.

**Disclosure:**

No significant relationships.

